# Skin sparing mastectomy and robotic latissimus dorsi-flap reconstruction through a single incision

**DOI:** 10.1186/s12957-019-1711-8

**Published:** 2019-11-02

**Authors:** Gilles Houvenaeghel, Marie Bannier, Sandrine Rua, Julien Barrou, Mellie Heinemann, Eric Lambaudie, Monique Cohen

**Affiliations:** 1 Department of Surgical Oncology, Paoli Calmettes Institute and CRCM, CNRS, INSERM, Aix Marseille Université, 232 Bd de Sainte Marguerite, 13009 Marseille, France; 2 Department of Surgical Oncology, Paoli Calmettes Institute, Marseille, France

**Keywords:** Breast reconstruction, Latissimus dorsi-flap, Robotic surgery

## Abstract

**Background:**

Robotic latissimus dorsi-flap reconstruction (RLDFR) after skin-sparing mastectomy (SSM) for breast cancer (BC) has been performed through a single nipple incision. We report results of SSM with RLDFR, mainly with analysis of feasibility, morbidity, indications, and technique standardization.

**Methods:**

We determined characteristics of patients, previous treatment of BC, and type of reconstruction. Surgical technique, duration of surgery, and complication rate were reported according to three successive periods: P1–3.

**Results:**

Forty RLDFR, with breast implant for 16 patients, with previous breast radiotherapy in 30% had been performed. In logistic regression, factors significantly associated with duration of surgery ≥ 300 min were P2 (OR 0.024, *p* = 0.004) and P3 (OR 0.012, *p* = 0.004) versus P1. The median mastectomy weight was 330 g and 460 g for BMI < and ≥ 23.5 (*p* = 0.025). Length of hospitalization was 4 days. Total complication rate was 20% (8/40): seven breast complications (four re-operations) and one RLDF complication with re-operation. Periods were significantly predictive of complications (*p* = 0.045).

**Conclusion:**

SSM with RLDFR is feasible, safe, and reproducible. We reported a decrease of duration of surgery, length of post-operative hospitalization, and complication rate.

## Introduction

Development of robotic surgery since several years was very important for prostatic cancer, gynecologic cancer, colo-rectal cancer, and thoracic and thyroid surgery [1–3]. Endoscopic non-robotic latissimus dorsi-flap breast reconstruction (LDFR) has been reported in several studies [4–9].

Very few experiences were reported in the field of breast surgery, with a small number of series including very few patients on robotic mastectomy or LDFR [10–14]. Nipple-sparing mastectomy (NSM) with immediate robotic latissimus dorsi-flap (RLDF) reconstruction has been reported in seven patients in Selber et al.’s study [10] and in four cases in Chung et al.’s study [12]. Skin-sparing mastectomy (SSM) with LDF reconstruction was reported in 17 patients for delayed-immediate breast reconstruction after SSM and placement of a tissue expander [13] and in one patient for immediate breast reconstruction with 3D endoscopy using another incision than areolar incision [15]. In a recent French prospective cohort study, Immediate breast reconstruction (IBR) was performed with LDFR in 46.9% of cases (24.3% combined with an implant), with implant in 46.5%, and rectus abdominis musculo-cutaneous flap in 6.6% [16]. The aim of this study was to report results of SSM with robotic LDFR performed during 29 months, through the analysis of feasibility, morbidity, indications, and standardization of patient positioning and operative technique.

## Material methods

A prospective cohort of patients undergoing SSM and robotic latissimus dorsi-flap reconstruction (RLDFR) over a period of 29 months (March 2016 to July 2018) was analyzed. All patients agreed to surgery with robotic assistance and received information on the procedure. The study protocol was approved by our institutional ethical committee.

We analyzed patient characteristics (age, body mass index (BMI), tobacco use, diabetes, ASA score, breast volume), previous treatment for breast cancer (BC) (sentinel lymph node biopsy (SLNB), axillary lymph node dissection (ALND), neo-adjuvant chemotherapy (NAC), previous breast radiotherapy), primary breast cancer (BC) or local recurrence, and type of reconstruction (LDFR with or without breast implant).

Surgical technique using da Vinci Si*®* Surgical System SI or XI (Intuitive Surgical, Sunnyvale, CA), number of trocars, skin incision, and duration of anesthesia and surgery were recorded according to period of treatment and associated surgical procedures (breast implant, LDFR, ALND, and contra-lateral breast surgery). Duration of anesthesia was defined as time from anesthesia induction to tracheal extubation and duration of surgery as time from skin incision to the end of skin suture including all associated procedures and changes in patient positioning. Three periods were established: P1 (year 2016), P2 (year 2017), and P3 (year 2018).

Complication rate was determined using Clavien-Dindo grading [17]. Re-operation rate, type of complication, and number of post-operative hospitalization days were reported.

### Statistics

Main characteristics were reported using median, mean, and 95% confidence interval (CI95) for quantitative criteria. Comparisons were performed using *χ*^2^ for categorical variables, *t* test or Anova for continuous variables, and logistic binary regression with odds ratios, CI95 and, *p* value with SPSS® software version 16.0. We considered *p* value ≤ 0.05 as significant result.

## Results

During the study period of 29 months, 119 patients were operated for breast surgery and/or RLDFR, 117 with da Vinci robot, and 2 with 3D endoscopy. Among these patients, we analyzed 40 patients with the same surgical procedure, SSM and RLDFR, performed by the same surgeon. Breast reconstruction was performed in 25 patients with autologous LDF associated with breast implant in 7 patients and in 15 patients with non-autologous LDF (without fat around LDF) associated with breast implant in 9 patients. The number of patients was 11, 18, and 11, respectively, for periods P1–P3. Chest sizes were 85, 90, 95, 100, and > 100, respectively, in 4, 10, 17, 6, and 3 patients. Patients’ characteristics are reported in Tables 1 and 2.

**Table 1:** Characteristics of all patients and according to periods of treatment

				P1-2016		P2-2017		P3-2018		*χ* ^2^
Population		*n*	%	*n*	%	*n*	%	*n*	%	*p*
Number patients		40		11	27.5	18	45.0	11	27.5	
Primary BC		35	87.5	7	63.6	17	94.4	11	100	0.017
Local recurrence		5	12.5	4	36.4	1	5.6	0	0	
Tobacco		9	22.5	5	45.5	3	16.7	1	9.1	0.090
Diabete		3	7.5	2	18.2	1	5.6	0	0	0.247
ASA	1	18	45.0	5	45.5	6	33.3	7	63.6	0.252
	2	21	52.5	5	45.5	12	66.7	4	36.4	
	3	1	2.5	1	9.1	0	0	0	0	
Breast size	A–B	15	37.5	2	18.2	8	44.4	5	45.4	0.20
	C	18	45.0	5	45.5	9	50.0	4	36.4	
	D–F	7	17.5	4	36.3	1	5.6	2	18.2	
Prosthesis size	< 300	5	29.4	4	40.0	1	16.7	0	0	0.50
	≥ 300	12	70.6	6	60.0	5	83.3	1	100	
Previous radiotherapy	Yes	12	30.0	6	54.5	4	22.2	2	18.2	0.10
	No	28	70.0	5	45.5	14	77.8	9	81.8	
Neo adjuvant chemotherapy		7	17.5	2	18.2	3	16.7	2	18.2	0.992
Reconstruction	Autologous LDF	18	45.0	2	18.2	12	66.7	4	36.4	< 0.0001
	Non-autologous LDF	6	15.0	0	0	0	0	6	54.5	
	LDF + implant	9	22.5	4	36.4	5	27.8	0	0	
	Autologous LDF + implant	7	17.5	5	45.5	1	5.6	1	9.1	
Incision for RLDFR	Axillar	7	17.5	3	27.3	3	16.7	1	9.1	0.490
	Areolar	33	82.5	8	72.7	15	83.3	10	90.9	
BC	Invasive	29	72.5	8	72.7	14	77.8	7	63.6	0.710
	DCIS	11	27.5	3	27.3	4	22.2	4	36.4	
Number of surgical procedures	2	21	52.5	2	18.2	12	66.7	7	63.6	0.070
	3	16	40.0	7	63.6	6	33.3	3	27.3	
	4	3	7.5	2	18.2	0	0	1	9.1	
da Vinci system	SI	17	42.5	11	100	6	33.3	0	0	< 0.0001
	XI	23	57.5	0	0	12	66.7	11	100	
Number of arms	2	29	72.5	3	27.3	15	83.3	11	100	< 0.0001
	3	11	27.5	8	72.7	3	16.7	0	0	
Hospitalization days	< 4 days	13	32.5	0	0	8	44.4	5	45.5	0.026
	≥ 4 days	27	67.5	11	100	10	55.6	6	54.5	
Time of surgery	< 300 mn	26	65.0	1	9.1	15	83.3	10	90.9	< 0.0001
	≥ 300 mn	14	35.0	10	90.9	3	16.7	1	9.1	
Time of anesthesia	< 382 mn	26	65.0	2	18.2	14	77.8	10	90.9	0.001
	≥ 382 mn	14	35.0	9	81.8	4	22.2	1	9.1	
BMI	< 23.5	17	42.5	5	45.5	7	38.9	5	45.5	0.916
	≥ 23.5	23	57.5	6	54.5	11	61.1	6	54.5	
Previous contra lateral BC	No	36	90.0	8	72.7	18	100	10	92.9	0.059
	Yes	4	10.0	3	27.3	0	0	1	9.1	
Previous homolateral BCT	No	21	52.5	5	45.5	10	55.6	6	54.5	0.859
	Yes	19	47.5	6	54.5	8	44.4	5	45.5	
Contra lateral breast surgery	No	38	95.0	11	100	17	94.4	10	90.9	0.613
	Yes	2	5.0	0	0	1	5.6	1	9.1	

*BMI* body mass index, *SI* da Vinci SI system, *XI* da Vinci XI system, *LDF* latissimus dorsi-flap, *NSM* nipple-sparing mastectomy, *RLDFR* robotic latissimus dorsi-flap reconstruction, *BC* breast cancer, *DCIS* ductal carcinomas in situ, *BCT* breast-conserving therapy

**Table 2:** Characteristics of patients and surgery

Population	All patients	Median	Mean	CI 95%	Range
Age		64.0	61.2	40.8–81.6	39–83
Mastectomy weight		358	445	345–546	72–1600
BMI		24.5	25.4	23.9–26.9	18.3–38.0
Duration of surgery		290	298	276–321	195–495
Duration of anesthesia		353	364	341–387	249–540
Hospital stay duration		4	4.38	3.89–4.86	2.0–8.0
Duration of surgery	P1	372	373	328–419	235–495
	P2	271	280	257–302	215–410
	P3	258	254	222–286	195–343
Duration of anesthesia	P1	428	438	393–483	313–540
	P2	355	351	328–373	276–457
	P3	323	313	281–345	249–404
Duration of surgery	SI	334	336	297–376	215–495
	XI	266	270	248–292	195–410
Duration of anesthesia	SI	403	410	376–444	313–540
	XI	331	330	307–353	249–457
Duration of surgery	2 surgical procedures	258	273	245–301	195–410
	3 surgical procedures	301	318	281–354	215–495
	4 surgical procedures	420	372	167–577	277–420
Duration of surgery	Autologous LDF	265	274	243–306	195–410
	Non autologous LDF	262	268	225–312	219–343
	LDF + implant	325	342	270–413	215–495
	Autologous LDF + implant	334	330	293–366	270–372
Duration of anesthesia	Autologous LDF	339	341	308–374	249–506
	Non-autologous LDF	327	327	276–378	252–404
	LDF + implant	409	412	351–474	294–540
	Autologous LDF + implant	403	394	353–435	342–451
Hospital stay duration	Autologous LDF	4	3.83	3.12–4.54	2.0–7.0
	Non-autologous LDF	3	3.33	2.79–3.88	3.0–4.0
	LDF + implant	5	4.89	3.99–5.79	3.0–7.0
	Autologous LDF + implant	6	6.0	4.81–7.19	4.0–8.0
BMI	Autologous LDF	25.3	26.6	23.8–29.4	18.3–38.0
	Non-autologous LDF	21.7	22.3	20.2–24.4	19.7–25.3
	LDF + implant	26.2	26.1	23.3–28.9	20.3–31.6
	Autologous LDF + implant	23.1	24.2	21.3–27.2	20.8–28.6
Mastectomy weight	Autologous LDF	340	503	298–708	100–1600
	Non-autologous LDF	225	238	77–400	72–423
	LDF + implant	370	409	281–536	201–696
	Autologous LDF + implant	488	492	342–641	263–778
Hospital stay duration	P1	6	5.64	5.02–6.26	4.0–7.0
	P2	4	3.83	3.19–4.48	2.0–7.0
	P3	4	4.0	2.88–5.12	2.0–8.0

*BMI* body mass index, *SI* da Vinci SI system, *XI* da Vinci XI system, *P* (1–3) period (1–3), *LDF* latissimus dorsi-flap

### Indications and type of reconstruction

Twelve patients had previous breast radiotherapy (30%, 12/40) including seven patients with SSM after NAC and radiotherapy (17.5%, 7/40). SSM was performed for five local BC recurrences with previous radiotherapy and 35 primary BC: 11 ductal carcinomas in situ (DCIS) and 29 invasive BC.

RLDFR with breast implant was performed in 40% of patients (16/40) (Table 3), in 58.3% (7/12) after previous radiotherapy. Mastectomy weight, breast cup size, and BMI according to the type of reconstruction are reported in Table 2.

**Table 3:** Results according to reconstruction type

		RLDF without implant		RLDF + implant		*χ* ^2^
		*n*	%	*n*	%	*p*
Age	≤ 50 years	8	33.3	3	18.8	0.261
	> 50 years	16	66.7	13	81.2	
Periods	P1	2	8.3	9	56.2	0.002
	P2	12	50.0	6	37.5	
	P3	10	41.7	1	6.2	
Breast cup size	A–B	12	50.0	3	18.8	
	C	9	37.5	9	56.2	
	≥ D	3	12.5	4	25.0	
BMI	< 23.5	10	41.7	7	43.8	0.576
	≥ 23.5	14	58.3	9	56.2	
Primary BC		23	95.8	12	75.0	0.073
Local recurrence		1	4.2	4	25.0	
Previous radiotherapy	No	19	79.2	9	56.2	
	Yes	5	20.9	7	43.8	
Hospitalization days	< 4 days	12	50.0	1	6.2	0.004
	≥ 4 days	12	50.0	15	93.8	
Duration of surgery	< 305 mn	20	83.3	6	37.5	0.004
	≥ 305 mn	4	16.7	10	62.5	
Duration of anesthesia	< 382 mn	19	79.2	7	43.8	0.025
	≥ 382 mn	5	20.8	9	56.2	
Mastectomy weight	≥ 330 g	12	52.2	4	25.0	0.085
	> 330 g	11	47.8	12	75.0	
		Median	CI 95%	Median	CI 95%	
Age		58	51–63	65	53–67	
Mastectomy weight		329	280–611	439	357–532	
BMI		24.0	23.5–28.0	25.6	23.4–27.1	
Hospitalization days		4	3.18–4.30	5	4.68–6.07	

*BMI* body mass index, *BC* breast cancer, *RLDF* robotic latissimus dorsi-flap

Robotic breast surgery was indicated in selected cases during the study period: 92 RLDF for IBR (40 SSM and 52 nipple sparing mastectomy) among 437 IBR (21%) and among 1193 patients who required a total mastectomy (7.7%). A selection of patients for RLDF was made according to patient’s wishes to avoid dorsal scar and to offer IBR without implant for patients who do not want implant breast reconstruction.

### Surgery: (Fig. 1)

All patients were either first positioned in dorsal decubitus for SSM followed by a side decubitus for RLDFR. The anterior border of the LD muscle and the inferior mammary fold were designed and marked before incision. Incision around the nipple areolar complex was performed for SSM, and LDF dissection was performed in 33 patients through this incision and in 7 patients through a short axillar incision more often during P1 and P2 (Table 1).

**Fig. 1:**
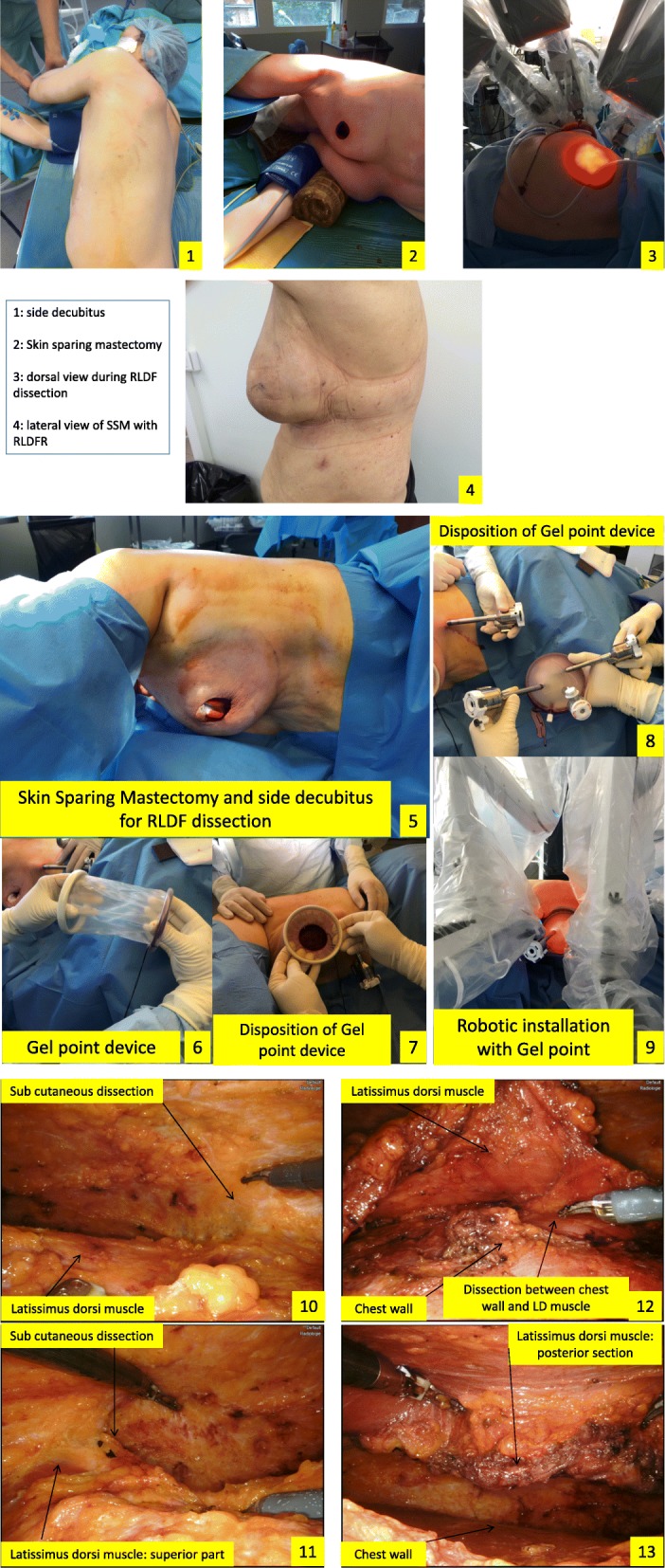
Surgical procedures

The beginning of the dissection for sub-cutaneous plan of LD muscle and a limited dissection under the incision along the anterior axillary line in order to introduced one robotic trocar about 6–7 cm under axillar basin (at the inferior mammary fold level) was performed.

Then, a Gelpoint*®* path single site device (Applied Medical) was inserted through the incision with two robotic trocars and one trocar for an Airseal*®* device insufflation (Applied Medical) also used by the assistant surgeon when necessary. We operated under low pressure (7 mmHg). Depending of the breast side, we inserted monopolar scissors and bipolar forceps into up and down robotic trocars with 0° camera in the middle robotic trocar.

Robotic surgery started with a superficial dissection of LD muscle from the middle of the muscle to the inferior part (5–6 cm under the inferior mammary fold) and to the superior part with a total section of the tendinous insertion. Then, we performed dissection underneath the LD muscle from the middle to the inferior part and to the level of vascular pedicle. The section of LD muscle was performed with monopolar scissors for posterior dorsal insertions, then at the inferior part of dissection, with progressive mobilization of muscle. Two drains were placed through the inferior infra-centimetric scar for the dorsal area and one for mastectomy.

Seventeen rights and 23 left SSM were realized. Robotic procedures were performed using SI daVinci system in 17 patients and XI system in 23 patients. We used 3 arms for 11 patients and 2 arms for 29 patients (72.5%): 3 arms 8/11 (72.37%) during 2016, 3/18 (16.7%) during 2017, and 0/11 during 2018 (*p* < 0.0001).

Concomitant with other surgical procedures, in 19 cases (47.5%), a previous partial ipsilateral breast resection had been performed. Axillary surgery was performed concomitantly in 23 cases (16 SLNB, 5 ALND, and 2 SLNB with ALND) (5 previous ALND for 5 local recurrences). A contra-lateral breast surgery was performed during the same operation in 2 (5%) patients.

### Duration of procedure

**Fig. 2:**
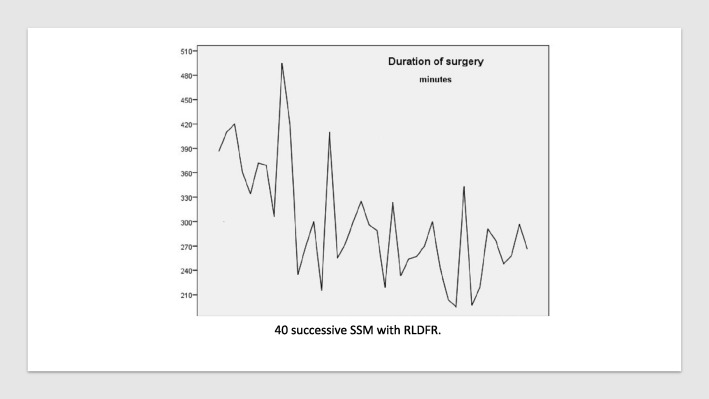
Duration of surgery for 40 successive patients in chronologic order

Median anesthesia duration was 353 min and median surgery duration was 290 min (Table 2). The duration of the surgery for the successive patients is reported in Fig. 2. The number of surgical procedures performed (LDFR, breast implant, ALND, contra-lateral breast surgery) was ≥ 3 for 19 patients (47.5%) including 3 patients with 4 procedures. BMI was ≥ 23.5 in 57.5% of patients (23/40).

In univariate analysis, duration of surgery were significantly different according to robot system used (*p* = 0.002), period P1 versus P2–3 (*p* < 0.0001 and non-significant between P2 and P3), number of robotic arms used (> for 3 arms: *p* < 0.0001), number of surgical procedures > 2 (*p* = 0.015) (non-significant for BMI < or ≥ 23.5: *p* = 0.790). In binary logistic regression including the 3 study periods and number of surgical procedures (> or ≤ 2), significant factors of duration of surgery ≥300mn were: P2 with a reduction in duration of surgery (OR 0.024, CI 0.002–0.298, *p* = 0.004) and P3 (OR: 0.012, CI 0.001–0.234, p = 0.004) versus P1 (number of surgical procedures: non-significant: *p* = 0.634). A strong correlation was observed between periods and robot system used and number of robotic arms.

In univariate analysis, the duration of anesthesia were significantly different according to the robot system used (*p* < 0.0001), number of robotic arms (> 3 arms, *p* < 0.0001), number of surgical procedures > 2 (*p* = 0.012), and period P1 versus P2–3 (*p* < 0.0001 and significance between P2 and P3, *p* = 0.043). In binary logistic regression including the three periods and the number of surgical procedures (> or ≤ 2), duration of anesthesia < or ≥ 382 mn differed significantly for the following periods: P2 (OR 0.062, CI 0.008–0.506, *p* = 0.009) and P3 (OR 0.022, CI 0.001–0.325, *p* = 0.006) versus P1 (number of surgical procedures—non-significant, *p* = 0.955).

Decrease rate of mean duration of surgery was 31.9% and decrease rate of mean duration of anesthesia was 28.5% from P1 to P3.

### Pathologic results

The median mastectomy weight was 401 g: 330 g (CI 251–377, mean 314, range 100–527) for BMI <  23.5 and 460 g (CI 378–696, mean 537, range 72–1600) for BMI ≥ 23.5, respectively (*p* = 0.025). Median breast implant volume was 340 cc (range 225–395).

The median size of invasive BC was 25 mm (mean 42.3, CI 28.6–56.1, range 0.7–130) with 15 multifocal BC (20 ductal, 10 lobular, 1 other type, and 9 DCIS). Median DCIS size was 50.0 mm (mean 54.2, CI 22.2–86.2, range 1–120).

#### Post-operative treatment

Six patients (21.4%) underwent post-mastectomy radiotherapy among 28 patients without previous radiotherapy, 8 patients received adjuvant chemotherapy, 28 patients endocrine therapy, and 3 patients received trastuzumab.

### Post-operative outcome

The median length of post-operative hospitalization was 4 days (Table 2): 13 patients < 4 days (32.5%) and 27 patients ≥ 4 days. Hospital stay ≥ 4 days was significantly associated with periods P1 versus P2–3 (11/11 for P1 and 16/29 for P2–3, *p* = 0.006), robot system used (*p* = 0.002), and type of reconstruction (*p* = 0.022). Others criteria analyzed were not significant: mastectomy weight, duration of anesthesia and surgery, previous radiotherapy (≥ 4 days 17/28 without and 10/12 with radiotherapy, non-significant), mastectomy for primary BC or local recurrence, BMI, age, and number of surgical procedures. In binary logistic regression, any factor was significant for post-operative hospitalization ≥ 4 days.

The total complication rate was 20% (eight patients): seven breast complications (three grade 1 and four grade 3: five hematomas, two infections) and one LDF complication (grade 3: dorsal bleeding). In univariate analysis, periods (P1, 5/11; P2, 2/18; and P3, 1/11) and robot system used (SI 5/17 and XI 3/23) were significantly associated with complications (respectively, 0.045 and 0.025), and all others factors were non-significant, particularly the type of reconstruction, BMI, and duration of surgery. Five re-operations (12.5%) were required (five grade 3): one for dorsal bleeding and four for breast complication (two hematomas and two infections with implant removal for one patient). Re-operations for dorsal bleeding and hematomas were made during hospitalization stay. For 14 patients, we observed dorsal seroma after drain removal that required one or several punctures. Patients were discharged before drain removal. Any conversion to an open technique for LDF dissection was required.

## Discussion

The purpose of this study was to assess feasibility of RLFR through a single incision around NAC required for SSM. The reproducibility of this procedure has been illustrated by no conversion to open technique, and a short axillar incision was used in 17.5% of patients particularly at the beginning of the experience. The safety of RLDFR has been also shown with only one complication for dorsal bleeding which required re-operation performed through the same incision (2.5%). We observed a significant decrease of the duration of surgery throughout the learning curve after the first period with 11 procedures. The mean duration of the whole procedure for the third period was 254 min and the robotic procedure currently lasts for approximately 45–60 min.

Very few experiences were reported for RLDF immediate breast reconstruction with no more than 17 procedures [9–12]. The main differences in robotic surgical technique that should be underlined included a single incision realized around NAC for SSM and the use of a single site trocar. In Selber et al.’s study [10], seven patients were reported with RLDF reconstruction performed through an axillar incision for NSM without the use of a single site trocar. Chung et al. [12] reported 12 RLDF procedures through a 5–6-cm axillar incision without CO2 gas insufflation for three delayed breast reconstructions, four IBRs with NSM, and five cases of chest wall deformity. Clemens et al. [13] reported 17 RLDFRs in delayed-immediate breast reconstruction after SSM and placement of a tissue expander through anterior mastectomy incision without a single site trocar.

Endoscopic non-robotic LDFR was reported in several studies [4–8], and in 2007, Missana et al. reported a study including 52 patients [4] and more recently by others with smaller series [6–8]. Nakajima et al. [8] reported a study with 168 LDF video-assisted reconstructions but only for reconstruction after partial mastectomy. Finally, Dejode and Barranger [15] reported one case of endoscopic 3D latissimus dorsi-flap harvesting for SSM with immediate breast reconstruction.

The endoscopic approach decreases donor-site morbidity [18] but the manual control of a two dimensional in-line endoscopic camera with limited internal mobility produces an inadequate optical window around the curvature of the thorax and the rigid-tip instruments also are inadequate to work along the curvature of the thorax. The use of 3D endoscopic surgery offers a magnified view but without the seven degrees of freedom of motion at the tips of the robotic instruments.

For patients with previous radiotherapy for local recurrence or after NAC and radiotherapy [19–21], the latissimus dorsi-muscle nourishes and protects the thin skin. In these cases, RLDFR can be associated with implant according to breast size and according to patient’s choice. One or several lipofillings were next proposed in order to obtain a good cosmetic result and sufficient breast volume. SSM was proposed for patients who want an IBR for whom NSM was not indicated (NAC involvement or tumor-NAC distance < 2 cm). Latissimus dorsi-flap reconstruction was offered in selected cases according to patient’s choice and particularly for patients who do not want reconstruction with breast implant (60% without implant in our study). More and more centers offer breast implant reconstruction with acellular dermal matrix (ADM). However, covering the entire implant with a thin, expensive ADM is not generally feasible, and the use of ADMs also increases the risk of complications such as infection and seroma [22].

## Conclusion

SSM with RLDFR is feasible, safe, and reproducible with a single incision for NAC resection. We reported with progressive learning curve a decrease of the duration of surgery, length of post-operative hospitalization, and complication rate. The robotic procedure currently lasts for approximately 45–60 min. Only one complication was related with RLDFR with re-operation for bleeding. After this technique standardization, we proposed to develop this procedure with several surgeons of our department using the double robotic console.

## Data Availability

Administrative data and clinical data are compiled in a common database and are available to editors and peer reviewers.
